# TET2: the fat controller of leptin

**DOI:** 10.1093/lifemeta/loae019

**Published:** 2024-05-24

**Authors:** Callen C Goldsmith, Garron T Dodd

**Affiliations:** Department of Anatomy and Physiology, The University of Melbourne, Melbourne, Victoria 3010, Australia; Department of Anatomy and Physiology, The University of Melbourne, Melbourne, Victoria 3010, Australia

**The mechanisms underlying leptin resistance in obesity remain incompletely understood. A recent publication in *Nature Communications* by Zeng *et al*. reports a previously undefined negative feedback loop involving ten-eleven translocation enzyme 2 (TET2) governing leptin expression and perpetuating leptin resistance in metabolic disease.**

Metabolic diseases such as obesity and type 2 diabetes have reached epidemic levels in developed nations like Australia, UK, and the USA [1]. In these countries, there are now more adults classified as overweight or obese than those maintaining a healthy weight [1]. At the core of these conditions lies energy homeostasis, an intricate equilibrium balancing the calories we consume and the energy we expend through metabolic processes, physical activity, and thermogenesis. Chronic imbalances in energy homeostasis, whereby energy intake exceeds expenditure, perpetuate the development and maintenance of metabolic disease. While this concept provides a foundational understanding, the mechanisms underpinning the progression of these diseases are highly complex and not fully understood.

Leptin is a hormone released from fat cells (adipocytes) [2], so it is secreted in direct proportion to the amount of energy that the body has stored as fat [3]. Leptin signaling in the hypothalamus of the brain enables the body to keep track of energy reserves, so it can alter the metabolic rate to maintain body weight homeostasis [3]. Exogenous leptin administration significantly reduces feeding behavior and increases energy expenditure in both rodents [4] and humans. Although it can reverse congenital leptin deficiency in these groups [5], it is ineffective in reversing diet-induced obesity. Intriguingly, individuals with diet-induced obesity show significantly elevated levels of circulating leptin and are hyperleptinemic [3]. This combination of hyperleptinemia with reduced leptin signaling and function is termed leptin resistance.

Significant research efforts have focused on the leptin signaling cascade to elucidate answers underpinning the hormonal resistance in obesity [4], however, this has led to underwhelming therapeutic traction. Recent research has pinpointed that hyperleptinemia, itself, may be a driving factor in leptin resistance as partially reducing circulating leptin levels using genetic and antibody-blocking methods improves leptin resistance [6], leading to decreased food intake, increased energy expenditure, and a reversal of obesity in humans and mice. The mechanisms and reasons behind exactly how reducing leptin levels in a hyperleptinemic state, like obesity, alleviate disease burden remain unknown.

To explore these mechanisms, a recent publication by Zeng *et al*., published in *Nature Communications*, investigated epigenetic factors underlying obesity, hyperleptinemia, and leptin resistance [7]. Epigenetics is a crucial mechanism linking environmental factors to obesity, as it allows for the regulation of gene transcription through DNA methylation and demethylation in response to environmental stimuli. The methylation of genomic DNA is performed by specialized enzymes called DNA-methyltransferases which catalyze DNA methylation by transferring a methyl to a 5ʹ cytosine in the context of cytosine-phosphate-guanine (CpG) to produce 5-methylcytosine (5-mC). In contrast, DNA demethylation is performed by ten-eleven translocation (TET) enzymes to convert 5-mC to 5-hydroxymethylcytosine (5-hmC). 5-hmC can then be further oxidized into other active forms before reverting to a normal cytosine during DNA replication. A recent study found that in adipose tissue alone (which secretes leptin), 2825 genes are associated with elevated body mass index (BMI) [8]. Moreover, in obesity, the methylation status of the adipocyte genome dramatically fluxes in response to changes in body weight [9]. Whilst a plethora of studies demonstrate clear evidence that DNA methylation occurs in the development of obesity, they fail to provide mechanistic or causal evidence as to whether DNA methylation contributes to obesity.

Exciting research suggests that the TET family of enzymes plays a key role in energy regulation within adipocytes. Deleting all three TET enzymes (TET1, TET2, and TET3) in fat cells protects against obesity during a dietary challenge by enhancing β-adrenergic-induced lipolysis and increasing adipose tissue thermogenesis [10]. *In vitro* studies also emphasize the role of TET2 in adipogenesis and insulin sensitivity in adipocytes through a peroxisome proliferator-activated receptor γ (PPARγ)-dependent mechanism [11]. However, the potential of TET2 modulation *in vivo* for treating metabolic disease remains unclear.

The study by Zeng *et al*. aimed to fill this gap, highlighting that decreased adipocyte TET2 levels are present in obesity which promotes downregulated 5-hmC. This opened the exciting question, as to how obesity drives changes in adipose tissue TET2 expression and function. To address this, Zeng and colleagues *et al*. reasoned that aberrant cellular signaling through a myriad of factors including proinflammatory cytokines, metabolic hormones, and pro-immune factors could be inhibiting TET2 production [7]. Utilizing a primary adipocyte culture, they showed that only leptin significantly reduced the levels of *Tet2* mRNA, an effect that could be restored by co-administration with a neutralizing leptin antibody. Using a small interfering RNA (siRNA) approach to inhibit leptin signaling through its classical Janus kinase2 (JAK2)/signal transducer and activator of transcription 3 (STAT3) signaling pathway, TET2 levels were restored in adipocyte cultures, collectively linking leptin signaling and the repression of *Tet2* expression in adipocytes.

Previous research into the role of TET2 in metabolic dysfunction revealed that reduced *Tet2* expression in adipose tissue endothelial cells promotes obesity by impeding vascularization and suppressing the browning of white adipose tissue [12]. In light of this understanding and given that *Tet2* expression is downregulated in obesity, one would intuitively hypothesize that adipose deletion of *Tet2* would exacerbate diet-induced obesity. Surprisingly, in both a whole body and adipose tissue-specific Cre recombinase mediated *Tet2* deletion, Zeng *et al*. showed a significant protective effect from high-fat diet-induced obesity. Both global and adipocyte-specific *Tet2* knockout lead to reduced weight gain and fat mass, improved insulin sensitivity, enhanced energy expenditure through elevated white and brown adipose tissue thermogenesis, decreased food intake, and decreased circulating leptin levels while consuming a high-fat diet. Previous work has revealed that leptin sensitivity can be restored in hyperleptinemic obesity by inhibiting leptin [6]. Therefore, mechanistically, decreased leptin levels facilitated by adipocyte *Tet2* deletion could reinstate leptin sensitivity and improve metabolic health. This theory is supported by Zeng *et al*. who showed that in adipose, *Tet2* knockout mice had stronger leptin-induced food intake suppression, which corresponded to a greater hypothalamic leptin signaling response assessed through elevated STAT3 phosphorylation. The key concepts of these results suggest that the positive effects of *Tet2* deletion is explicitly linked to reduced leptin expression, preventing a hyperleptinemic state during high-fat diet feeding. To mechanistically define the protective element of *Tet2*-adipocyte knockout in obesity, wild-type (WT) and adipocyte *Tet2* deficient mice were fed a high-fat diet, however, *Tet2*-adipocyte knockout mice were given daily doses of leptin to create an artificial hyperleptinemic state analogous to WT mice. Intriguingly, this prevented any of the protective benefits associated with adipocyte *Tet2* deletion, identifying a mechanistic link suggesting that *Tet2* adipocyte expression drives hyperleptinemia and leptin resistance.

This raises the question of how TET2 coordinates leptin expression in adipocytes. To address this, Zeng *et al*. performed chromatin immunoprecipitation sequencing (ChIP-seq), a technique used to identify genome-wide DNA binding sites for transcription factors. They found a TET2 spike in the leptin promoter region near the transcription initiation sequence of leptin. The role of TET2 in leptin expression was then confirmed through enzymatic inhibition of TET2 in a mature primary adipocyte culture, resulting in significantly reduced leptin gene transcription into mRNA. Furthermore, adipocytes of *Tet2* deficient mice exhibited decreased levels of 5-hmC and elevated 5-mC, methylation within the leptin gene. These results suggest a link between TET2 and leptin expression, however, TET2, unlike TET1 or TET3, lacks a DNA binding motif and, therefore, will require binding partners to engage with the leptin gene. The ChIP-seq data revealed that within the TET2 peak at the leptin promoter, there are binding motifs for three different transcription factors: specificity protein 1 (SP1), CCAAT enhancer-binding protein α (C/EBPα), and activator protein-2β (AP-2β). Zeng *et al.* reasoned that one of these factors may be the binding partner of TET2. AP-2β is a known negative regulator of leptin, therefore it’s unlikely to be TET2’s binding partner to enable leptin expression, and therefore it was excluded [7]. Utilizing siRNA, they independently knocked down the other two candidates, SP1 and C/EBPα, but only *Cebpa* knockdown disrupted the TET2 peak on the leptin gene promoter. Moreover, co-immunoprecipitation experiments on mature adipocytes displayed a physical interaction between TET2 and C/EBPα. Finally, through ChIP and sequential ChIP (ReChIP) assays, it was shown that TET2 and C/EBPα are recruited to the leptin gene promoter. Collectively, Zeng *et al*. open an exciting new area of discovery within the epigenetic modulation of energy homeostasis and its role in the development of obesity [7].

The pre-clinical work by Zeng *et al*. suggests that leptin self-regulates its expression by suppressing TET2 levels, and this system is augmented in obesity leading to hyperleptinemia. An important outstanding question is whether a similar feedback loop occurs in humans. Recapitulating the pre-clinical data, they showed that subcutaneous adipose tissue from obese patients had significantly reduced 5-hmC and elevated 5-mC levels. Moreover, the obese patients (BMI 37.2 ± 3.8 kg/m^2^) had significantly reduced *TET2* levels and higher *LEPTIN* expression than non-obese patients (BMI 23.1 ± 1.3 kg/m^2^). The levels of *TET2* were negatively associated with both *LEPTIN* and BMI and enzymatically inhibiting TET2 in mature human adipocytes decreased leptin production. These clinical results support the pre-clinical data, suggesting that similar mechanisms in obesity-related hyperleptinemia are present in humans.

**Figure 1:**
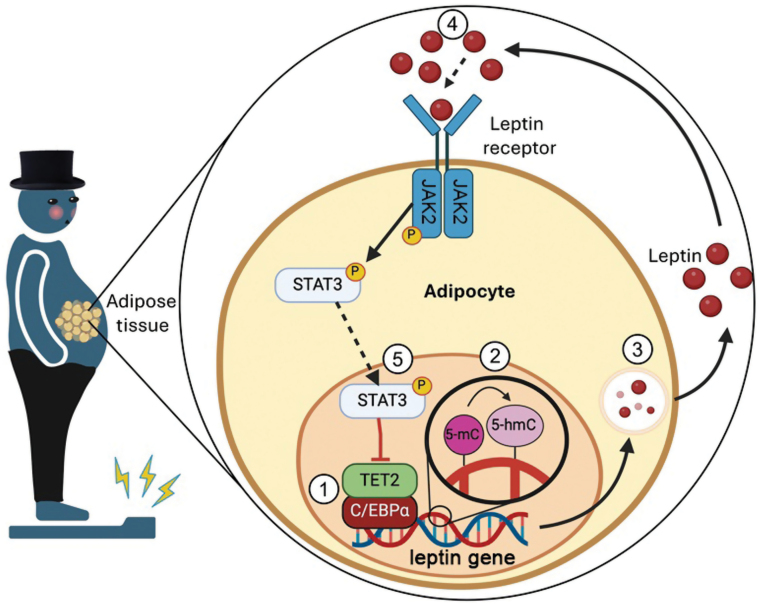
Adipocyte *Tet2* expression perpetuates a leptin signaling feedback loop. (1) TET2 forms an association with C/EBPα, enabling it to bind with the leptin promoter and (2) catalyze the conversion of 5-mC to 5-hmC. (3) This promotes leptin expression, where leptin is exported out of the cell. (4) Through autocrine or paracrine signaling, leptin signals via its receptor expressed on adipocytes via JAK2/STAT3 where (5) it inhibits TET2. Created with BioRender.com.

Conceptually, Zeng *et al*. unveil a novel negative feedback loop that regulates leptin expression. They demonstrate that TET2 interacts with transcription factor C/EBPα to demethylate the leptin gene, promoting leptin expression, and leptin then indirectly inhibits its own expression through autocrine signaling by inhibiting the expression of *TET2* through JAK2/STAT3-dependent signaling (Fig. 1). This innovative research broadens our understanding of how leptin is regulated and provides a novel mechanism that could be targeted to treat obesity. Several intriguing unanswered and compelling questions arise from the research by Zeng *et al.*, e.g., if TET2 is endogenously suppressed in obesity, how are obese patients present in a hyperleptinemic state? An important experiment left out of the current paper is what the methylation state of the leptin promoter is in obesity. The expression of leptin is governed by many factors, especially the hormone insulin [2]. Therefore, there is a possibility that while TET2 is suppressed, the leptin promoter remains demethylated and leptin is expressed through a secondary unexplored pathway.

Previous work in accordance with Zeng *et al*. shows that deletion of all TET enzymes from adipose tissue protects from high-fat diet-induced obesity by enhancing β-adrenergic signaling leading to increased fatty acid oxidation, and thermogenesis [10]. However, work emerging on adipocyte-adjacent endothelial cells in obesity reveals that increasing their TET2 levels induces similar effects on energy expenditure in adipocytes through TET2’s modulation of endothelial-secreted bone morphogenetic protein 4 [12]. Therefore, it is intriguing that the global *Tet2* deletion by Zeng *et al*. resulted in analogous effects to adipocyte-specific *Tet2* deletion, since there is such heterogeneity in TET2’s effects [7, 10, 12] even within the same tissue niche. Moreover, TET enzymes govern expression over thousands of genes within adipose tissue alone, not discounting other organs. Therefore, the effects of *Tet2* deletion or suppression will be broad throughout the genome. Pursuing this line of reasoning presents an exciting new area of study since the function of TET2 across multi-organ systems remains an emerging field with much to be deciphered. Especially, different cell types respond with high variability to *Tet2* knockdown as previously discussed with endothelial versus adipocytes [7, 10, 12]. A final question arises about the capacity of TET2 modulation to treat obesity. Zeng *et al*. and others have shown that altering the expression of *Tet2* can promote a healthier phenotype under a high-fat diet challenge [7, 10, 12]. However, it remains unexplored whether this same TET2 modulation can improve the phenotype of an already obese individual. Collectively, Zeng *et al*. open an exciting new area of discovery within the epigenetic modulation of energy homeostasis and its role in the development of obesity.
